# Patent foramen ovale in an elderly male with multiple embolic infarcts

**DOI:** 10.1002/ccr3.1852

**Published:** 2018-10-26

**Authors:** Sander Johan Aarli, Jostein Kråkenes, Tom Roar Omdal, Ulrike Waje‐Andreassen

**Affiliations:** ^1^ Department of Neurology Haukeland University Hospital Bergen Norway; ^2^ Department of Clinical Medicine University of Bergen Bergen Norway; ^3^ Department of Radiology Haukeland University Hospital Bergen Norway; ^4^ Department of Heart Disease Haukeland University Hospital Bergen Norway

**Keywords:** embolism, neurology, patent foramen ovale, stroke, ultrasound

## Abstract

Patent foramen ovale (PFO) is associated with embolic stroke, particularly in younger patients. In older patients, atrial fibrillation is a more common cause of embolic stroke. A PFO may be considered culpable at higher age when other potential embolic sources are absent or after recurrent stroke despite prophylactic treatment.

## 1 CASE REPORT

**Figure 1:**
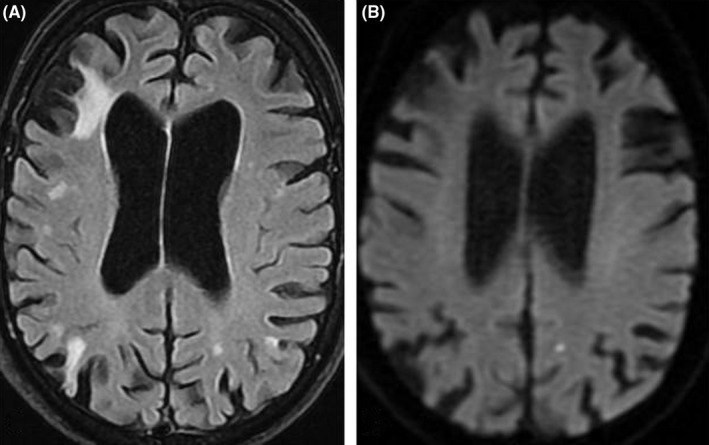
MRI on current admission. A, Multiple cortical and subcortical infarct sequelae on MRI FLAIR. B, Acute microinfarct on diffusion‐weighted MRI (DWI)

**Figure 2:**
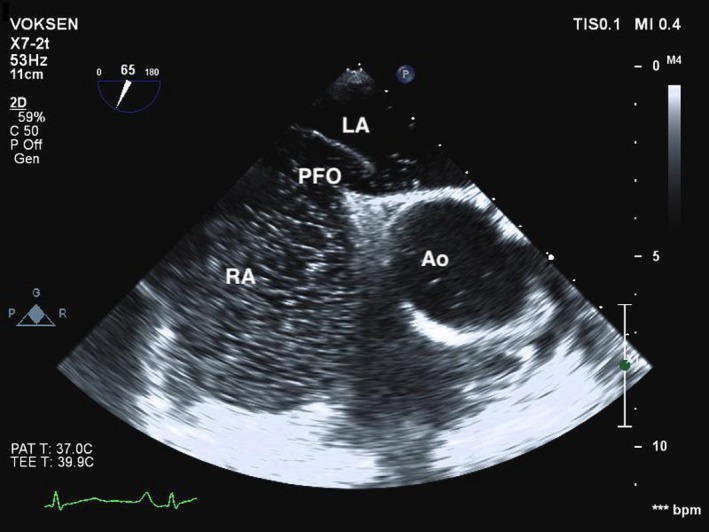
Transesophageal echocardiography verified a large PFO. Ao, aorta; LA, left atrium; PFO, patent foramen ovale; RA, right atrium

An 85‐year‐old Caucasian male was admitted after sudden onset of expressive aphasia and weakness in both legs lasting 20 seconds. He was athletic, self‐reliant and had no cognitive impairment. During the last 28 years, he had experienced 8‐10 heterogeneous episodes of acute neurological symptoms, such as central facial palsy, hemiparesis, and non‐fluent aphasia, lasting from seconds to 3‐4 hours. Precerebral duplex and electrocardiography (ECG) were performed several times with normal results, and EEG registration and 24‐hour Holter monitoring had been normal. Previous MRI scans showed no abnormal restricted diffusion, as seen in acute cerebral infarcts, but infarct sequelae in the left temporal lobe and both thalami. Several years later, three additional infarct sequelae were detected in the cerebellum. The patient was treated with platelet inhibitors, and medications and dosages were adjusted after new episodes. There was no suspicion of lack of compliance. Except from age, migraine, and previous smoking, with cessation 35 years ago, he had no known risk factors for cerebrovascular disease. On the current admission, he presented with reduced motor speed in his left arm and leg. Electrocardiography and Holter monitoring showed no signs of atrial fibrillation. CT and MRI revealed multiple, cortical infarct sequelae in the anterior and posterior circulation territories of both hemispheres, and MRI also detected two acute embolic infarcts in the right occipital lobe and one in the left parietal lobe (Figure 1). CT and MRI angiograms and duplex sonography did not show significant plaques or stenoses, and pre‐ and intracerebral flow were normal with asymmetrical vertebral arteries, which were considered a normal anatomical variant. Cortical infarcts in several vascular territories strongly suggest cardioembolic etiology, but transthoracic echocardiogram showed no cardiac sources of emboli, and there was no sign of left atrial enlargement, which may be seen in the presence of atrial fibrillation. The patient concurred to further diagnostic tests aiming to determine the cause of recurrent cerebral emboli, although he was informed that the results would not necessarily alter treatment recommendations. We performed a transcranial Doppler (TCD) bubble test with 10 mL air‐mixed saline injected into the left cubital vein while the left middle cerebral artery was insonated with a 2‐MHz probe. Injection at resting state produced no microembolic signals, while injection after Valsalva maneuver resulted in a shower of microembolic signals followed by single signals persisting for over 30 seconds. The result implied the presence of a latent right‐to‐left shunt, and transesophageal echocardiography verified a large patent foramen ovale (PFO; Figure 2). In agreement with the patient, we decided on non‐operative treatment. Due to previous failure of antiplatelet treatment, we changed to a direct oral anticoagulant (dabigatran 110 mg twice daily), intended as a lifelong treatment. He had no subjective complaints at discharge.

## 2 DISCUSSION

Patent foramen ovale is a potential interatrial shunt present in 25% of the adult population.^1^ Several observational studies describe increased prevalence in young patients with ischemic, cryptogenic stroke, but a significant association is also seen in older stroke patients.^2^, ^3^ The hypothesized mechanism is paradoxical embolization by shunting from venous to arterial circulation. Despite large data, causality is still disputed due to high prevalence of PFO, low recurrence rate of clinical ischemic stroke and possible overestimation of PFO in patients with cryptogenic stroke.^4^ Venous thromboembolism is more frequent among older patients,^5^ and the same is likely for Valsalva maneuvers, due to increasing incidence of constipation, prostatic problems, and obstructive sleep apnea with higher age. As Valsalva maneuvers promote right‐to‐left shunting, it is possible that a PFO would result in more paradoxical embolization with higher age. Yet, in older patients, the competing causes of stroke are more frequent, which reduce the probability of a PFO being culprit. In addition, older patients seldom undergo the special diagnostic tests necessary to detect a PFO. The diagnostic standard test is transesophageal echocardiography; however, the TCD bubble test has good diagnostic accuracy for detecting a right‐to‐left shunt. Due to its simplicity and non‐invasive nature, it is considered a feasible first choice for PFO screening.^6^, ^7^ When assessing the clinical relevance of a PFO, it is crucial to exclude other causes of stroke, and thus identify true cryptogenic infarcts. Our patient had a positive TCD bubble test, and transesophageal echocardiography confirmed a large PFO. The diagnostic evaluation revealed no other plausible causes, and we considered paradoxical embolization to be the most likely cause of multiple cerebral emboli through 28 years. A hypothetical alternative would be subclinical atrial fibrillation not detected by Holter registrations. Yet, after several embolic strokes over decades, we would expect atrial fibrillation to be more persisting and thereby apparent on Holter registrations. Several multicentre randomized controlled trials (RCTs) have compared device closure and medical treatment for PFO. The first trials (CLOSURE I, PC, and RESPECT) did not show benefit of device closure, although secondary analyses and long‐term data from the RESPECT trial suggested a reduced risk of embolic events after closure.^8^, ^9^, ^10^, ^11^ The Gore REDUCE trial and CLOSE trial applied stricter inclusion criteria by excluding patients with a high burden of vascular risk factors, thus increasing the probability of identifying true cryptogenic infarcts. Both trials showed a significant benefit of device closure.^12^, ^13^ Recent meta‐analyses with pooled data from all five RCTs concluded that device closure is superior to medical treatment alone for preventing stroke recurrence in selected patients with cryptogenic infarcts; however, none of the studies included patients over the age of 60.^14^, ^15^ In the analysis by Lattanzi and colleagues, age did not influence the benefit of closure, while Reinthaler and colleagues found a greater benefit among patients under the age of 45. Atrial fibrillation is the most common adverse event after PFO closure, and the rate of adverse events is higher in older patients.^16^ A clinical practice guideline by Kuijpers and colleagues concludes that the applicability of the RCT conclusions is uncertain for patients over 60 years, and the guideline authors expect that PFO closure in older patients would lead to smaller benefits and greater harms.^17^ We therefore found non‐operative treatment more advantageous for this 85‐year‐old patient with sinus rhythm and no subjective complaints but changed from platelet inhibitors to a direct oral anticoagulant. Anticoagulation would also be an adequate treatment for possible undetected atrial fibrillation, although, we considered this a less probable cause of the recurrent embolic stroke lesions over decades. Risk factors change throughout life, and snoring, causing increased intrathoracic pressure similar to Valsalva maneuver, may have led to repeated and summarized paradoxical embolization to the cerebral cortex in our patient. We conclude that paradoxical embolization should be considered at higher age when usual diagnostic tests are negative, and patients are interested in, and receptive to, further investigations. Atrial fibrillation is not always the cause of embolic stroke, and PFO may also be culpable at higher age. Nevertheless, oral anticoagulation may be a common treatment option for both conditions.

## DISCLOSURES

All authors report no disclosures.

## AUTHOR CONTRIBUTION

SJA: involved in concept and design, collection of data and drafting, and revision of the manuscript. JK and TRO: collected, analyzed and interpreted the data and revised the manuscript. UWA: involved in concept and design, collection, analysis, and interpretation of data and revision of the manuscript.
